# Mutations Associated to Antibiotic-Resistant *Helicobacter pylori*: It Is Time to Validate Sequencing Observations

**DOI:** 10.3390/jcm11154293

**Published:** 2022-07-24

**Authors:** Maxime Pichon, Victor Luzarraga, Christophe Burucoa

**Affiliations:** 1 CHU Poitiers, Bacteriology Laboratory, Infectious Agents Department, 86021 Poitiers, France; victor.luzzaraga@chu-poitiers.fr (V.L.); christophe.burucoa@chu-poitiers.fr (C.B.); 2 Pharmacology of Antimicrobial Agents and Antibiotic Resistance, University of Poitiers, INSERM U1070, 86034 Poitiers, France

*H. pylori*, a Gram-negative microaerophilic microorganism, is the only bacterial pathogen classified as a Class I carcinogen, due to its association, since its initial discovery, with severe gastric disease (including gastritis, gastric ulcers, gastric adenocarcinoma, or lymphoid tissue lymphoma associated with mucosa), and its extraordinary overall prevalence, estimated at 4.4 billion patients worldwide [1,2]. It is estimated that 15% of the infected patients will develop gastric diseases associated with this infection.

As *H. pylori* is a primary cause of gastric cancer, with a high mortality rate, immense resources are devoted to the development and research of treatment for *H. pylori* infections. Treatment is key to patient management, as *H. pylori* is responsible for histopathological cascades to cancer, which have been reported in large-scale epidemiological studies and reinforced by in vitro and in vivo studies. Furthermore, these data suggest that eradication of the bacterium may halt or even reverse carcinogenesis. Most infected patients receive empirical treatment with a proton pump inhibitor combined with dual antibiotics (tetracycline–metronidazole) or even triple antibiotics (amoxicillin–clarithromycin–metronidazole) combined with bismuth salts or dual antibiotics (amoxicillin–clarithromycin) without bismuth salts. However, the level of eradication failure is increasing due to a rise in resistance to first-line antibiotics, particularly clarithromycin. In some regions, where culture or PCR methods predominate, the rates of primary or secondary resistance to clarithromycin, metronidazole and quinolones increase over time, reaching levels that do not allow non-guided antibiotic therapy according to recommendations [3]. These unacceptable levels have justified the classification of this bacterium in the list of high-priority antibiotic-resistant bacteria [4].

As the main genetic supports of antibiotic resistance have been “clearly” identified (*plp1* for amoxicillin; 16S rDNA for cyclins, 23S rDNA for clarithromycin, *frxA/rdxA* for metronidazole, *gyrA/B* for quinolon, *rpoB* for rifamycins), the democratization of nucleotide sequencing techniques (Ultra-deep Sequencing) coupled with the development of bioinformatics have recently favored investigations aimed at analyzing these bacterial populations [5]. Nevertheless, the level of evidence of the association of these mutations with the development of antibiotic resistance is not always very high, as it relies on sequencing by very varied approaches, without always including controls attesting to the quality of the process. In view of the very large number of mutations that have been associated in vitro/in vivo/in silico with antibiotic resistance, it is time to set up not only retrospective, but also prospective validation of a database compiling the mutations with a real impact and that should be searched for before treatment, following the example of the WHO’s data curation for *Mycobacterium tuberculosis*.

Since, to our knowledge, no such database has yet been effectively compiled, we are proposing the validation criteria necessary to consider a newly discovered mutation as solidly proven. First, the discovery must be observed on an isolated bacterial strain rather than on a clinical sample, whether or not the patient is in therapeutic failure. If the mutation cannot be found, the authors should obtain the strain and perform the susceptibility test according to the current reference procedure. If observed in a resistant strain, the mutation cannot be considered “sufficient to produce an antibiotic-resistant strain” if it is also observed in an antibiotic-sensitive strain [6]. This point could be discussed when additional mutations are needed to obtain a significantly resistant strain. In this case, the mutation may be considered necessary, but not sufficient (and should be considered as such) if the presence of mutation is associated with a significant (four-fold) increase in the minimum inhibitory concentration (MIC). Second, MIC determination must be achieved using a validated procedure. This procedure should be standardized according to current recommendations, i.e., for EUCAST 2022 recommendations, using Mueller–Hinton agar with 10% horse blood (or 5% horse blood defibrinated with 20 mg/L β-NAD, e.g., MHF). The inoculum should be evaluated at a concentration of 3McFa (after checking for the absence of coccoid forms) and be incubated after plating in a microaerobiose atmosphere for 48 to 72 h at 35+/−2 °C. For the EUCAST recommendation, resistance thresholds have been defined epidemiologically and should help to compare strains from international studies.

From our point of view, the best validation of the impact of a genetic mutation in terms of antibiotic resistance could be based on transformation approaches. Helicobacter pylori is a naturally transformable bacterial strain that could easily be transformed using validated approaches described long ago (since 1998) by Wang and Taylor [7]. This procedure allows a reference (antibiotic-susceptible) Helicobacter pylori strain to be transformed so as to include any mutation, with the scientist having only to design four appropriate primers. Validation of the transformation is further accomplished by producing antibiotic-containing agar (containing *a minima* an antibiotic concentration reflecting the threshold considered significant for defining an antibiotic-resistant strain) on which the putative antibiotic-resistant strains will grow. Sequencing of the cultured strains must be performed so as to validate their transformation and to confirm the absence of compensatory mutation. In the absence of culture on resistant agar, sequencing and/or specific PCR should be performed on the entire subpopulation in order to observe and/or quantify the transformed subpopulation. This will enable definitive classification of the impact of the mutation on antibiotic resistance. In addition, special attention should be paid to the genetic background of the selected strain, the objective being to ensure that there are no mutations associated with geographic-specific antibiotic resistance. To resolve this problem, the authors could use the most appropriate reference strain according to the geographical location of the patient from whom the resistant strain was isolated, or else use two or more reference strains, thereby validating the impact of the mutation on the genetic background [8].

Finally, it is important to emphasize that for diagnosis, at least two different biopsies, in the antrum and the fundus, are required. This is justified by the heterogeneity of the distribution of resistant bacterial subpopulations, which may nonetheless be present throughout the tract [9]. In addition, mutations associated with antibiotic resistance appear to be present at different frequencies in different regions of the stomach, once again justifying the analysis of at least two different regions to ensure robust results. Stool PCR globalizing subpopulations could help to detect minor variant subpopulations [10,11].

In conclusion, the increasing rate of antibiotic resistance is a major public health problem, especially in major bacterial pathogens such as *Helicobacter pylori*. In such infections, it is widely recognized that guided antibiotic therapy is crucial to optimize clinical management. In the context of difficult bacterial culture and given the democratization of molecular biology systems in medical laboratories, validated databases developed in appropriate models to guide the development of new antibiotic molecules or diagnostic procedures are crucial prior to further exploration in animal model examples. The proposed approach will open a new field of research on therapeutic development designed to render therapies more effective by improving the initial choice of antibiotics. The results achieved will improve the clinical and fundamental knowledge of *Helicobacter* infections themselves.
